# A conversation about building a support network in science

**DOI:** 10.1038/s41467-023-36599-6

**Published:** 2023-02-23

## Abstract

Organizations have been founded to build communities by bringing together scientists from diverse backgrounds but with one shared identity and the common goal of strengthening their roles, increasing their visibility, and promoting their representation. In this conversation, representatives from three such organizations share their experiences and advice with *Nature Communications*. *Priscilla Kolibea Mante* (a Co-Chair), *Encieh Erfani* (a member of the Executive Committee) and *Lisa Herzog* (an alumna) of the Global Young Academy (GYA) discuss the role of their organization in supporting early career researchers. *Kaela Singleton*, the president-elect of Black In Neuro, discusses their mission to empower Black neuroscientists. *Jennifer Thomson*, the president of the Organization for Women in Science for the Developing World (OWSD), informs us about their efforts in providing career development, networking and leadership opportunities to women from the developing (and developed) world.

1. What is your organization about and how did you begin to engage with it? How has it evolved over time, also with regards to membership numbers and their expectations?

**GYA:** The Global Young Academy (GYA) is a vibrant network that endeavors to provide a voice for early career scholars. By providing training on leadership skills to its members, the GYA enables early career researchers (ECRs) to tackle universal challenges through evidence-based science diplomacy. What began as a 2010 workshop of 40 young scholars from 28 countries who had a vision of giving a voice to young scholars around the world, has evolved into a robust independent science academy with a capped membership of 200 current members to maintain the dynamics of the academy and an ever-growing number of alumni representing 100 countries. Membership at the GYA is determined through an application process with a deadline on the 15th of September each year, with selection based on the excellence and impact of the applicants.

**Black In Neuro:** Black In Neuro is a registered 501(c)(3) organization founded in 2020 by seventeen Black and six non-Black neuroscientists from various career stages. Our organization and my engagement with Black In Neuro came together from a single tweet from the current president Dr. Angeline Dukes and has expanded from there. Our mission is to diversify the neurosciences by building a community that celebrates and empowers Black scholars and professionals in neuroscience-related fields. This celebration of Blackness within the neurosciences blossomed with a viral promotional video (seen over 140,000 times on Twitter), millions of engagements via Twitter and Instagram and the collation of over 300 profiles of Black neuroscientists on Blackinneuro.com. The success and reach of Black In Neuro has led us to go about becoming a permanent fixture within the world of neuroscience and STEM and push towards diversity, equity and inclusion, representation, accountability, and justice within academia.

**OWSD:** The Organization for Women in Science for the Developing World (OWSD) is an international organization, which aims to unite eminent women scientists from the developing, as well as the developed, worlds with the objective of strengthening their role in professional development and promoting their representation in scientific and technological leadership. It provides research training, career development, and networking opportunities for women scientists throughout the developing world at different stages in their careers, including PhD fellowships, Early Career fellowships and prizes. Networking, ensuring recognition of women in science, and helping younger women enter and thrive in science are among our priorities. I joined OWSD soon after I co-founded the South African Women in Science and Engineering in 1996, and I was elected OWSD President in 2016 and re-elected in 2021. The backbone of our organization are the 44 National Chapters, which are based in our four regions: Latin America and the Caribbean, Africa, the Arab countries and Asia plus the Pacific.

**Figure:**
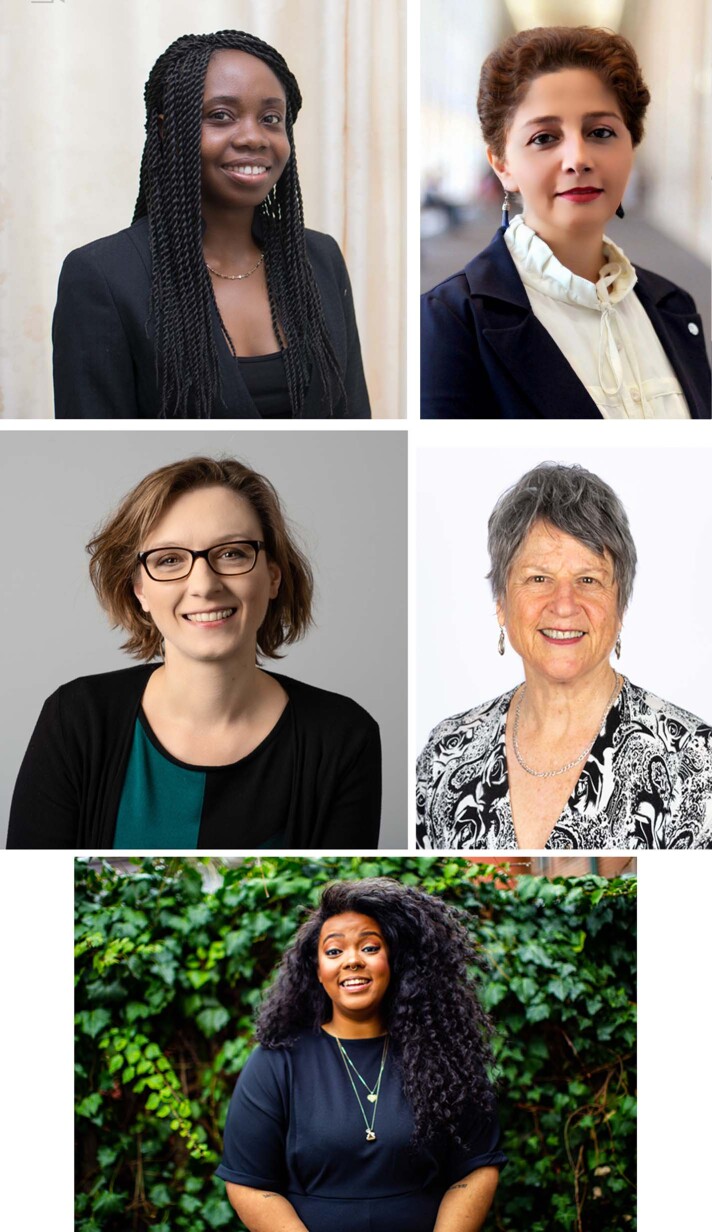
Top left: Priscilla Kolibea Mante is Co-Chair of the Global Young Academy, a neuroscientist and epilepsy drug expert at the Kwame Nkrumah University of Science and Technology in Kumasi, Ghana. Top right: Encieh Erfani has been a member of the Executive Committee of the Global Young Academy since 2021 and is an assistant professor of Cosmology at the Institute for Advanced Studies in Basic Sciences in Iran. Middle left: Lisa Herzog is an alumna of the Global Young Academy and Dean of the Faculty of Philosophy at the University of Groningen in the Netherlands. Middle right: Jennifer Thomson is President of the Organization for Women in Science for the Developing World and Emeritus Professor in the Department of Molecular and Cell Biology at the University of Cape Town in South Africa. Bottom: Kaela Singleton is president-elect of Black in Neuro as well as a National Institute of Neurological Disorders and Stroke and Burroughs Wellcome Postdoctoral Enrichment fellow, completing her training in the Faundez lab at Emory University in the United States. Priscilla Kolibea Mante; Matthew Jordaan; Sylvia Germes; Jennifer Thomson; Mathew White.

2. How do you advocate for your members? What activities, both big and small, do you plan in the short- (1–2 years) and long-term (5–10 years)? Why are these your priorities?

**GYA:** The GYA runs several projects advocating for ECRs. One of our flagship projects, the Global State of Young Scientists (GloSYS), highlights the challenges young researchers face in Asia, Africa, Latin America, and the Caribbean, including work instability, limited research budgets, and balancing work with family time. Our At-Risk Scholars Initiative gives a voice to and supports refugee and displaced researchers worldwide, and supports the re-integration of these scholars into research through GYA networks. Further, our Science Advice project provides advice for policy makers specifically in relation to shaping policy affecting young scientists. As the GYA Constitution notes, we support the exchange of ideas across disciplines, cultures, nationalities, and generations, which can expand opportunities for scientists, increase scientific capacity and lead to meaningful change around the world. These continue to be our guide posts for all of our activities.

**Black In Neuro:** As Black In Neuro has grown, so has the way we advocate for our members. In our inception in 2020, visibility and expressing shared experiences were the top ways we ensured our community was valued and supported. Since then, we’ve progressed and in addition to those metrics, we’ve provided resources, tools, and opportunities to share with our community, such as our seminar series, sponsored panels, and professional development workshops as well as a new mentorship program.

**OWSD:** I take every opportunity I can to talk or write about OWSD, whether that’s at the Hegra Conference of Nobel Laureates, the annual South African Science Forum or the African Science Summit, or through my recent book “Travels with my Lab Notebook”, in which I dedicate one chapter to women in science. Our priorities in the short term are (1) Educating about sexual harassment in science, (2) Setting up fruitful interactions with the scientific diaspora (women working in developed countries), (3) Increasing our presence in countries where we are poorly represented, and (4) Helping women in mid-careers get ahead. In the longer term, we plan to call on members who are men to strengthen relationships with women in science beyond the OWSD, plus obtain funding for our National Chapters.

3. What has been your biggest achievement in building a community? Why do you think this is your biggest achievement?

**GYA:** As we are now active members of international organizations at the science policy interface, we have hopefully lived up to the founding GYA members’ ideals. The GYA is recognized and accepted by the international science community as a valued contributor to the InterAcademy Partnership and the International Science Council, which we consider an important achievement as we are the only young academy among their members. Additionally, we convened our own panel at the Science Summit at the 77th UN General Assembly Science Summit.

**Black In Neuro:** This is probably one of the tougher questions. I’m confident each Black In Neuro member has their own ‘biggest achievement’ moment that speaks to the specific sense of belonging and community the organization has built. Our first annual conference was a huge milestone and success for both early career scientists and the organizing team. Seeing Black scholars talk about their research was empowering. Additionally, every Black In Neuro week holds a special place in each organizer’s heart. Seeing updates from various scholars whether that be life or career updates and watching them succeed and achieve their dreams year after year has to be some of the most rewarding work we’ve ever done.

**OWSD:** Being actively involved in the lives of our National Chapters (NCs) and getting our Vice-Presidents and Regional Members to do so as well. These include our active participation in NC launches and workshops, when traveling is possible. In addition, encouraging NCs to link up with other organizations in their countries, such as National Academies. In this way, they can tap into other networks and receive help, financially and in kind. These types of assistance enable NC members to expand their networks, as well as to promote OWSD within their countries.

4. What has been the biggest challenge in building a community? How has this challenge been overcome/do you think it could be overcome in the future?

**GYA:** Organizations such as GYA thrive on the interactions between community members for growth. Identifying unique scholars who want to create societal impact by working closely with other members, while ensuring the assembled network is diverse and inclusive, can be difficult. The membership selection process is therefore crucial. GYA members are selected through a process that is very responsive to changing times and circumstances and has evolved over the years to establish a thoroughly diverse and inclusive network that has, for example, a majority of female members – a rarity among international science organizations. Another issue is that member engagement during the pandemic was challenging to maintain, because we could not meet in-person as we normally do at our Annual General Meetings, which have migrated online for the last three years. We therefore feel great enthusiasm for our planned in-person Annual General meeting and Conference in 2023 in Kigali, Rwanda.

**Black In Neuro:** One of the biggest challenges in building Black In Neuro is ensuring every voice is heard. From global perspectives to varying identities, we want every Black person within the field regardless of age, sexual identity, gender etc. to be supported and feel valued. Our community calls to action, requests for feedback, and surveys conducted via social media are prime examples of how we do that, as well as making ourselves available to other early career scientists for mentorship and guidance.

**OWSD:** My biggest challenge in building a community has been funding. We have very limited funding for anything other than our PhD and Early Career Fellowships, and the OWSD/Elsevier Foundation prizes, as well as for our secretariat. I am hoping to overcome this by interacting more with the many organizations who attended our 2021 General Assembly online and in person. I intend to follow up the attendees’ interest and enthusiasm in the months to come, especially as international travel becomes easier.

5. What are the top three suggestions you would give to someone looking to start an organization similar to yours?

**GYA:** (1) Establish a purpose for the organization: It is important to think about why this organization is needed, what you want to achieve with it and what this organization would contribute to society. Focusing on a purpose will help you establish the fundamental principles that will define the model of the organization. (2) Identify allies: This will help you garner support for the establishment of the organization. (3) Establish a process for selecting members and active member engagement: Firm criteria for member selection are important. Membership is key to achieving the vision of the organization.

**Black In Neuro:** Ensure you work on a team that has shared missions and values. Give people the space to be their authentic selves. Lastly, communicate!

**OWSD:** There are three recommendations that I would like to pass on: (1) Get together a group of people who are as passionate about their aims as you are, and who are prepared to put in considerable amounts of time in achieving these. (2) Set your priorities at a realistic level, especially in the beginning. (3) Look for champions who can advocate for your organization to larger audiences.

6. Is there anything else you would like to share?

**GYA:** One project we have recently completed in science communication is SCISO, which stands for “Science with Society“. SCISO grew out of discussions in our Trust in (Young) Scientists working group about how to build trust between science and society, and what we, as young scientists, can do to help bridge the gap between science and society. SCISO features videos, with subtitles in various languages, about the role of science in society, practical tips for science communication, and best practice examples of science communication and collaborations with the public.

**Black In Neuro:** Building Black In Neuro and engaging with the community has been an incredible experience. Even as president-elect, Black In Neuro has given me community and belonging over the past two years that I never had in my prior ten years of experience in the field. I’m incredibly honored to take over the organization in summer 2023 and I hope I can continue to provide that sense of mutual support and give back to a community that’s given me so much confidence and courage.

**OWSD:** Yes—I would like to highlight the importance of the incredibly dedicated and hard-working OWSD Secretariat, without whom we could not function. Generally speaking, I would like to acknowledge that organizations like ours rely on a healthy collaborative environment.

