# Publisher Correction: A conversation about building a support network in science

**DOI:** 10.1038/s41467-023-37803-3

**Published:** 2023-04-11

Correction to: *Nature Communications* 10.1038/s41467-023-36599-6, published online 23 February 2023

The original version of this Article contained an error in the caption to the profile photos, which incorrectly read ‘Priscilla Kolibea Mante is Co-Chair of the Global Young Academy, a neuroscientist and epilepsy drug expert at the Kwame Nkrumah University of Science and Technology in Kumasi, Ghana.’ The correct version states ‘Top left: Priscilla Kolibea Mante is Co-Chair of the Global Young Academy, a neuroscientist and epilepsy drug expert at the Kwame Nkrumah University of Science and Technology in Kumasi, Ghana. Top right: Encieh Erfani has been a member of the Executive Committee of the Global Young Academy since 2021 and is an assistant professor of Cosmology at the Institute for Advanced Studies in Basic Sciences in Iran. Middle left: Lisa Herzog is an alumna of the Global Young Academy and Dean of the Faculty of Philosophy at the University of Groningen in the Netherlands. Middle right: Jennifer Thomson is President of the Organization for Women in Science for the Developing World and Emeritus Professor in the Department of Molecular and Cell Biology at the University of Cape Town in South Africa. Bottom: Kaela Singleton is president-elect of Black in Neuro as well as a National Institute of Neurological Disorders and Stroke and Burroughs Wellcome Postdoctoral Enrichment fellow, completing her training in the Faundez lab at Emory University in the United States.’

The original version of this Article contained an error in the response to question 6 by the GYA, which incorrectly read ‘One project we have recently completed in science communication is SCISO, which stands for “Science with Society“.’ The correct version states ‘One project we have recently completed in science communication is SCISO, which stands for “Science with Society [https://globalyoungacademy.net/sciso/]”.’

This has been corrected in both the PDF and HTML versions of the Article.

